# Auditory evoked potential could reflect emotional sensitivity and impulsivity

**DOI:** 10.1038/srep37683

**Published:** 2016-12-02

**Authors:** Ji Sun Kim, Sungkean Kim, Wookyoung Jung, Chang-Hwan Im, Seung-Hwan Lee

**Affiliations:** 1 Clinical Emotion and Cognition Research Laboratory, Inje University, Goyang, Republic of Korea; 2 Department of Biomedical Engineering, Hanyang University, Seoul, Republic of Korea; 3 Department of Psychiatry, Inje University, Ilsan-Paik Hospital, Goyang, Republic of Korea

## Abstract

Emotional sensitivity and impulsivity could cause interpersonal conflicts and neuropsychiatric problems. Serotonin is correlated with behavioral inhibition and impulsivity. This study evaluated whether the loudness dependence of auditory evoked potential (LDAEP), a potential biological marker of central serotonergic activity, could reflect emotional sensitivity and impulsivity. A total of 157 healthy individuals were recruited, who performed LDAEP and Go/Nogo paradigms during electroencephalogram measurement. Barratt impulsivity scale (BIS), Conners’ Adult ADHD rating scale (CAARS), and affective lability scale (ALS) were evaluated. Comparison between low and high LDAEP groups was conducted for behavioural, psychological, and event-related potential (ERP) measures. The high LDAEP group showed significantly increased BIS, a subscale of the CAARS, ALS, and false alarm rate of Nogo stimuli compared to the low LDAEP group. LDAEP showed significant positive correlations with the depression scale, ALS scores, subscale of the CAARS and Nogo-P3 amplitude. In the source activity of Nogo-P3, the cuneus, lingual gyrus, and precentral gyrus activities were significantly increased in the high LDAEP group. Our study revealed that LDAEP could reflect emotional sensitivity and impulsivity. LDAEP, an auditory evoked potential could be a useful tool to evaluate emotional regulation.

Individual personality traits such as emotional sensitivity and impulsivity could cause social conflicts, which can undesirably be manifested in criminal behavior or violence^1^. In addition, emotional dysregulation and impulsive behavior are deeply involved with various neuropsychiatric problems^2^. To verify the aversive issues related to emotional sensitivity and impulsivity, researchers have attempted to evaluate the associated traits.

Loudness dependence of the auditory evoked potentials (LDAEP) was calculated as the amplitude change of the evoked N1/P2 component in response to different auditory stimulus intensities^3^. LDAEP has been identified to be inversely associated with central nervous system serotonergic activity^4^, and has been proposed as a reliable indicator of central serotonin activity in humans^5^. In clinical studies, Fitzgerald *et al*.^6^ reported that MDD patients with melancholic features (i.e., lack of mood reactivity) had a significantly weaker LDAEP slope, whereas our previous study showed stronger LDAEP values in atypical depression^7^. While, previous studies reported that individuals who were sensitive to external stimuli have stronger emotional responses^8^, the specific relationship between emotional sensitivity and LDAEP has not been clarified yet.

Meanwhile, impulsivity has been defined as the lack of ability to refrain inappropriate behavior and clinically, has been regarded as the inability to inhibit behavioral response^9^. Recent meta-analysis reported that the components of event-related potential (ERP) of Go/Nogo tasks were associated with response inhibition^10^. For example, Nogo-N2 has been suggested to reflect a variety of cognitive control processes that underlie response inhibition, including response activation^11^, premotor inhibition^12^, and most importantly, conflict monitoring^13^; Nogo-P3 has been proposed to primarily reflect the inhibitory process itself^11^. In the Nogo trials, the P3 component, has been linked with the process of response inhibition^14^. Especially, N2 and P3 components, and accuracy rate from the Nogo task have mainly changed in patients with impulse control problems such as trichotillomania, antisocial personality disorder and Attention Deficit Hyperactivity Disorder (ADHD)^15^,^16^,^17^. Both Nogo-N2 and P3 ERP component have been regarded to reflect impulsivity, however the interpretation of the changes in N2 and P3 ERP is inconclusive^18^. Furthermore, previous studies revealed that poor performance of Nogo could be related to low serotonin function and genetic mutation of serotonin system^19^,^20^, suggesting that serotonin function might be related with Nogo trials and that serotonin might play a core role among behavioral inhibition and LDAEP^21^. Previous studies directly pointed out that LDAEP was stronger in more impulsive individuals^22^. Despite the plausible relationship between LDAEP and impulsivity reflected by Go/Nogo paradigm, there have been no studies investigating the association between LDAEP and Nogo ERP in the general population.

Hence, we hypothesized that individuals with high LDAEP would demonstrate higher emotional sensitivity and impulsivity (Fig. 1). The first aim of the present study was to verify the relationship between LDAEP and emotional sensitivity measured by psychological scaling. The second aim of the study was to evaluate the relationship between LDAEP and impulsivity (impulsivity rating scales and Nogo-N2, P3). Finally, we explored the regional activity of the brain through source activity analysis of the Nogo ERP. It would support our hypothesis by verifying that brain regions known to be related with response inhibition activate in coherence with the change of the Nogo ERP.

## Results

### Psychological and Behavioural measures

Table 1 displays comparisons of the demographic and psychological characteristics between participants in the low and high LDAEP groups. The women *vs*. men ratio of the high LDAEP group was higher, than that of the low LDAEP group (*p* = 0.01). Scores of the Barratt impulsivity scale (BIS) (attentional impulsivity & motor impulsivity), Conners’ Adult ADHD rating scale (CAARS) (impulsivity/emotional lability), and affective lability scale (ALS) (depression/elation & anger) were significantly greater in the high LDAEP group compared to the low LDAEP group. Beck depression inventory (BDI) and ALS (anxiety/depression) were marginally significantly greater in the high LDAEP group compared to the low LDAEP group.

**Figure 2:**
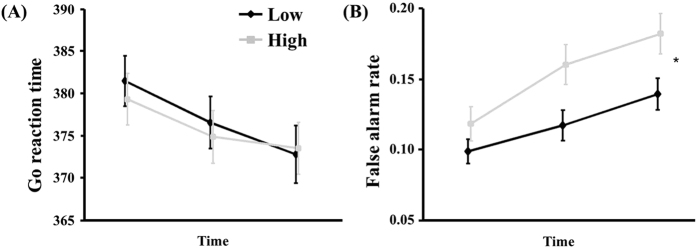
(**A**) The reaction time of Go between the low and high loudness dependence of the auditory evoked potential (LDAEP) groups. (**B**) The false alarm rate of Nogo between the two groups. The x-axis of each figure denotes the duration of Go/Nogo task. The mean and standard error of the mean are presented. * represents a statistically significant difference between the two groups (*p* < 0.05).

There was no significant difference in the average reaction time (376.55 *vs.* 375.65 ms, *F* = 0.910, df = 1, *p* = 0.34), and hit rate (0.94 *vs.* 0.93 ms, *F* = 0.075, df = 1, *p* = 0.78) in the Go condition between the low and high LDAEP groups. However, there was a significant difference in the false alarm rate of Nogo stimuli between the two groups (0.11 *vs.* 0.15, *F* = 6.184, df = 1, *p* = 0.01) (Table 1, Fig. 2).

### Electroencephalogram Data

**Figure 3:**
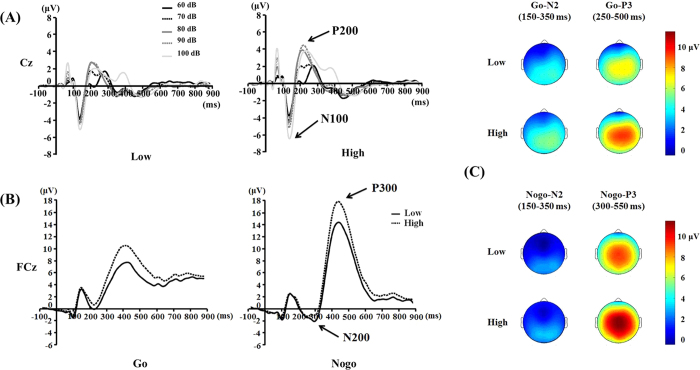
(**A**) Grand averages of loudness dependence of the auditory evoked potential (LDAEP) event-related potentials (ERPs) at the Cz electrode for the low and high LDAEP groups. (**B**) Grand averages of Go ERPs and Nogo ERPs at the FCz electrode between the low and high LDAEP groups. (**C**) Scalp topographies of Go/Nogo N2 and P3 components between the two groups.

Figure 3(A) presents the LDAEP waveforms at Cz in low and high LDAEP groups. Figure 3(B) presents the Go and Nogo ERP waveforms at FCz electrodes.

#### Loudness dependence auditory evoked potentials (LDAEP)

The subjects were divided into two subgroups based on the median LDAEP (=0.98) at the Cz electrode: the low group (n = 78, 0.56 ± 0.37) and the high group (n = 79, 1.53 ± 0.43). The peak-to-peak N1/P2 amplitudes for the five sound intensities of the low LDAEP group were 60 dB: 6.61 ± 2.24; 70 dB: 7.12 ± 2.55; 80 dB: 8.13 ± 2.60; 90 dB: 8.28 ± 2.52; 100 dB: 8.82 ± 2.59 μV, and the peak-to-peak N1/P2 amplitudes for the five sound intensities of the high LDAEP group were 60 dB: 6.89 ± 2.84; 70 dB: 8.10 ± 2.83; 80 dB: 9.84 ± 3.24; 90 dB: 11.14 ± 3.42; 100 dB: 13.00 ± 3.28 μV.

#### Go/Nogo condition

Because there were no group differences for latencies of N2 and P3 components across the Go and Nogo stimuli, the following analysis focused on the amplitude of each component.

In the N2 amplitude, there was a significant main effect of condition (*F* = 21.477, df = 1, *p* < 0.001). The main effect of electrode site was also significant (*F* = 12.681, df = 3, *p* < 0.001). Post-hoc analysis revealed that the N2 amplitudes of Fz and FCz were greater (more negative) than those of Cz and Pz. Importantly, the two-way interaction of group x condition was significant (*F* = 7.456, df = 1, *p* = 0.007). However, post-hoc analysis revealed that there was no significant difference between two groups in both Go and Nogo conditions.

In the P3 amplitude, there was a significant main effect of group (*F* = 10.838, df = 1, *p* = 0.001). The main effects of condition and electrode site were significant (*F* = 23.163, df = 1, *p* < 0.001; *F* = 9.866, df = 3, *p* < 0.001, respectively) as well. Post-hoc analysis revealed that the P3 amplitudes of FCz and Cz were greater than Fz and Pz. Although there was no significant interaction, all the three factors (group, condition, and electrode site) showed significant main effect and a simple main effect analysis was performed to check if there was a group difference in the Go and Nogo conditions. The simple main effect analysis indicated that both amplitudes of Go-P3 and Nogo-P3 were significantly lower in the low LDAEP group than in the high LDAEP group regardless of the electrode sites.

### Correlation analysis

**Figure 4:**
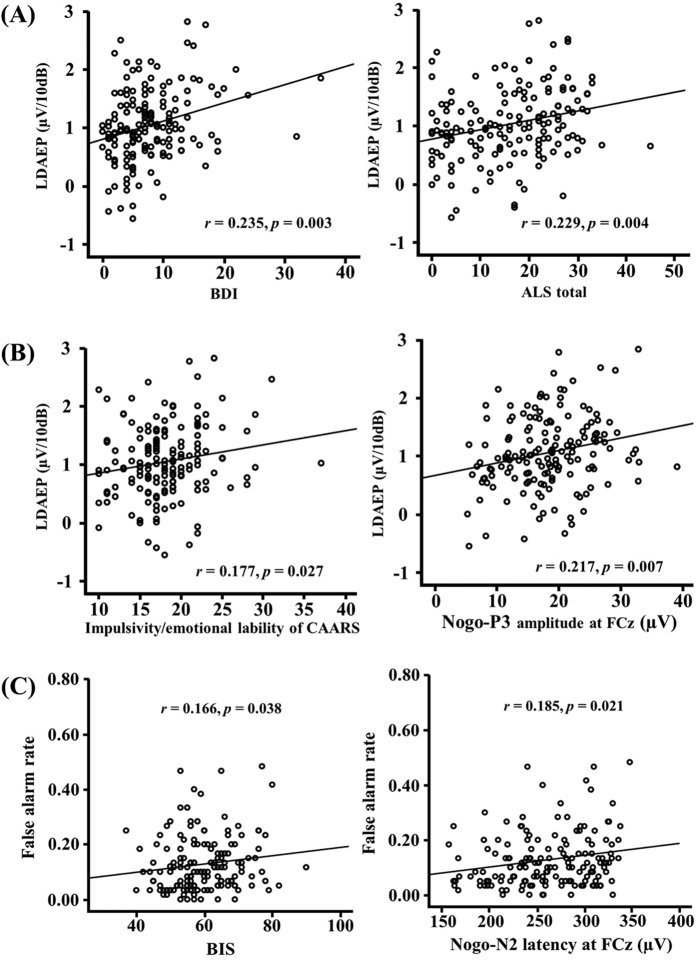
(**A**) The loudness dependence of the auditory evoked potential (LDAEP) showed a significant correlation with emotional scale scores such as Beck Depression Inventory (BDI), and Korean version of Affective Lability Scale (ALS) scores. (**B**) LDAEP showed significant correlation with impulsivity measures such as impulsivity/emotional lability subscale score of adult attention-deficit hyperactivity disorder (ADHD) scale, and Nogo P3 amplitude at FCz. (**C**) The false alarm rate of Nogo showed a significant correlation with impulsivity measures such as Barratt Impulsivity Scale (BIS) score and Nogo-N2 latency at FCz.

LDAEP was significantly correlated with psychological measures related to emotionality such as BDI (r = 0.235, *p* = 0.003), ALS total score (r = 0.229, *p* = 0.004) (Fig. 4A), the depression/elation subscale (r = 0.248, *p* = 0.002) and the anxiety/depression of the ALS (r = 0.175, *p* = 0.029), and anger (r = 0.165, *p* = 0.041). The previous studies revealed that the FCz electrode showed the greatest P3 amplitude and stronger N2 correlation compared to those of other electrodes^23^,^24^. In addition, the FCz showed robust findings in both N2 and P3 amplitudes in this study. Based on these findings, for the correlation between LDAEP and ERP, the FCz electrode was used for analysis to avoid the multiple comparisons. LDAEP was also significantly correlated with impulsivity measures such as impulsivity/emotional lability (r = 0.177, *p* = 0.027) of CAARS and Nogo-P3 amplitude at the FCz (r = 0.217, *p* = 0.007) (Fig. 4B). In addition, the false alarm rate of Nogo condition showed a significant positive correlation with the total score of BIS (r = 0.166, *p* = 0.038), and Nogo-N2 latency at the FCz electrode (r = 0.185, *p* = 0.021) (Fig. 4C).

### Source P300 of Nogo condition

**Figure 5:**
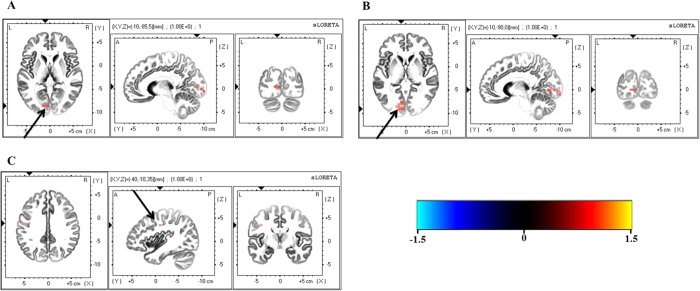
Differences in the source activity of Nogo P300 between the low and high loudness dependence of the auditory evoked potential (LDAEP) groups in three regions: (**A**) cuneus, (**B**) lingual gyrus, and (**C**) precentral gyrus.

Source analysis of the Nogo-P3 revealed increased source densities of the cuneus (BA 17), lingual gyrus (BA 17, 18), and precentral gyrus (BA 6) in the high LDAEP group (*p* < 0.05; Fig. 5) compared to the low LDAEP group. Detailed information on the statistical values and voxel coordinates is provided in Table 2.

## Discussion

This study investigated whether nonclinical adults with higher LDAEP show higher emotional sensitivity and higher impulsivity. First, the high LDAEP group showed higher emotional sensitivity measured by psychological scaling. Second, the high LDAEP group showed increased Nogo false alarm rate, and increased BIS score compared to the low LDAEP group. The LDAEP values were significantly correlated with BIS score and Nogo-P3 amplitude. Additionally, the source activity of the Nogo P300 revealed significantly greater activation of the cuneus, lingual gyrus, and precentral gyrus in the high LDAEP group compared to those in the low LDAEP group.

As we hypothesized, the LDAEP values were correlated with BDI and ALS. The high LDAEP group also showed higher depressive symptom scores and affective lability than the low LDAEP group. Our results show that the level of LDAEP is closely related with emotional sensitivity, and reflect “mood reactivity or fluctuation tendency” in healthy participants. Similarly, a previous clinical study revealed that LDAEP is significantly related to the mood reactivity in patients with major depressive disorder^25^. These evidences suggest that LDAEP might be a marker for the emotional sensitivity not only in the clinical condition, but also in the general population.

The BIS scores of the high LDAEP group was higher than that of the lower group. The high LDAEP group showed significantly lower accuracy rate for Nogo trials as well. Additionally, the false alarm rate of Nogo was positively correlated with the BIS in this study. In previous studies, the false alarm (commision error) rate of Nogo has been known to be closely related to response inhibition^26^,^27^ and also associated with trait impulsiveness as measured by BIS^18^,^28^. These evidences suggest that high LDAEP would demonstrate higher impulsivity related to impulsivity scale and false alarm rate for Nogo trials.

Interestingly, post hoc revealed no significant difference between the two groups for both Go and Nogo conditions in the Go/Nogo-N2 amplitude, even though there was a significant two-way interaction of group x condition. However, we found that the degree of amplitude changes from Go condition to Nogo condition was larger in the high LDAEP group than in the low LDAEP group; the high LDAEP group showed lower Go-N2 amplitude and increased Nogo-N2 amplitude than the low LDAEP group. Smaller amplitude of Go-N2 may mirror decreased attention resources allocated to the competing stimuli^17^. Considering that Nogo-N2 is widely accepted to be relevant to conflict monitoring^13^, this increased Nogo-N2 amplitude in the high LDAEP group might reflect a compensatory mechanism that prevents less efficient conflict monitoring. Therefore, the increased degree of amplitude changes between Go and Nogo conditions in the high LDAEP group might indicate that less attention resource would be allocated to the competing stimuli and this would lead to weaker conflict monitor in the high LDAEP group than in the low LDAEP group.

As we expected, LDAEP was positively correlated with Nogo-P3 amplitude and Nogo-P3 amplitude was significantly higher in the high LDAEP group compared to the low LDAEP group. Previous studies suggest that the Nogo-P3 amplitude is related to impulsivity^16^,^29^. Hartman *et al*.^26^ insisted that diminished Nogo-P3 might be an indicator for poor response inhibition. Nogo-P3 components showed reduction of the amplitudes in juvenile delinquents with antisocial personality characteristics^17^, patients with ADHD^16^, and borderline personality disorder^30^. In contrast, people with internet addiction disorder exhibited higher Nogo-P3 amplitude than controls^29^. The increased Nogo-P3 in non-clinical individuals with high impulsivity reflect the need for enhanced inhibitory effort or the degree of cognitive endeavors in order to yield equal performance compared to that of low impulsive individuals^14^,^29^,^31^. Moreover, Benvenuti *et al*.^18^ commented that high impulsive individuals may require an greater effortful response inhibition in order to counteract the prepotent tendency to respond, which is elicited by the combination of high trait impulsiveness and high emotional arousal. This increased P3 amplitude might reflect a protective or compensatory mechanism that prevents the premature response or poor impulse control in high LDAEP group.

People with high sensory processing sensitivity are described to reflect an increased sensitivity of the central nervous system and a deeper cognitive processing of physical and emotional stimuli^32^. Considering that the higher LDAEP might be related to higher sensory sensitivity, these individuals could better respond to the positive and negative stimuli with higher reactivity. As a result, higher reactivity to negative stimuli could cause depressive mood or anxiety related to emotional sensitivity. On the other hand, the more sensitive individuals, with heightened positive emotions in response to rewarding stimuli, might be associated to the “openness” on the five basic personality dimension^33^.

Meanwhile, a previous study using functional magnetic resonance imaging reported that healthy subjects with higher impulsivity have a similar influence on the neuronal correlates of the coding of sound intensity and show more activation of auditory evoked potential than subjects with low impulsivity^34^. In the study, high impulsive subjects are presumed to show a greater serotonergic responsiveness, representing lower levels of serotonin, causing a higher auditory evoked activity in the primary auditory cortex, which is correlated with the loudness-dependent change of the extent of fMRI activation^32^. The underlying mechanism has not been clarified yet, LDAEP in the primary auditory cortex is positively correlated to “novelty seeking”^35^ and it might be related to impulsive choice related to the serotonergic responsiveness.

In the source activity of Nogo ERP, the precentral gyrus (BA 6), lingual gyrus and cuneus showed stronger activation in the high LDAEP group compared to the low LDAEP group. The precentral gyrus is related to motor control and plays a critical role in inhibiting inappropriate prepotent response tendencies in motor process^36^,^37^,^38^,^39^. The lingual gyrus is one of the activated regions in error processing and response inhibition in healthy controls^37^,^40^. The Left cuneus is one of the activated regions during response inhibition task in healthy control as well as patients with schizophrenia^41^,^42^. Activation of these regions during response inhibition task could be interpreted as an increased demand for more inhibitory efforts and resources. It suggests that the high LDAEP group need more effortful impulse control compared to the low LDAEP group.

There are some limitations in this study. First, the gender ratio was different between the high and low LDAEP groups. To overcome gender effects, we used partial correlation in LDAEP related analysis. Secondly, the present study lacked structured interview that screen healthy participants. Finally, our results may not be generalized to the clinical subjects. Further studies would be needed to evaluate pathophysiology of the clinical samples.

To our knowledge, this is the first study to show the relationship between LDAEP and Go/Nogo ERP reflecting impulsivity in non-clinical participants of large sample sizes. Consistent with our hypothesis, LDAEP was associated with emotional sensitivity and impulsivity. The source analysis of Nogo ERP supports our hypothesis. These evidences suggest that LDAEP is a useful tool to evaluate emotional regulation such as emotional sensitivity and impulsivity in healthy individuals.

## Method

### Participants

This study was approved by the Institutional Review Board and Ethics Committee of Inje University Ilsan Paik Hospital and all experimental protocols were approved by the committee (2015-07-026-001). The study was performed in accordance with approved guidelines. Informed consent was obtained from all study participants. The study was performed on 157 non-smoking healthy volunteers (57 men and 100 women) with a mean age of 27.80 ± 6.37 (years). Participants were recruited from the local community through local newspapers and posters. Participants with any history of neurological or other mental diseases, and smoking history within 2 years were excluded from the study through the initial screening interviews. Each participant had normal or corrected to normal vision, as determined by checking visual acuity with the Snellen chart^43^.

### Psychological measures

Psychological measures and scales were conducted to measure emotional sensitivity and impulsivity. To evaluate emotional sensitivity, Beck Depression Inventory (BDI)^44^ State-Trait Anxiety Inventory (STAI)^45^ and Korean version of Affective Lability Scale (KALS)^46^,^47^ were applied. BDI is a validated scale composed of 21-items for measuring the severity of depressive symptoms^44^. Each BDI question was scored from 0–3, with higher scores indicating greater depressive symptom severity. The State-Trait Anxiety Inventory (STAI) is a commonly used tool that measures trait and state anxiety^45^. It includes the state anxiety inventory (SAI) and the trait anxiety inventory (TAI), which are comprised of 20-items each^45^. The 18-item ALS, which measures individual proneness to rapid shifts from the different emotional states of anxiety, depression, anger, and hypomania^46^, was also evaluated. The ALS is based on a three factor model of affective lability (depression/elation, anxiety/depression, and anger)^48^.

To assess impulsivity related traits, Barratt Impulsiveness Scale (BIS)^49^,^50^, Conners’ Adult ADHD rating scale (CAARS)^51^, Behavioral Activation System, and Behavioral Inhibition System^52^,^53^ were applied. The BIS consists of 11 questionnaires and is designed to assess the personality/behavioural construct of impulsiveness. The BIS has three second-order factors (attentional, motor, and non-planning impulsiveness)^50^. The CAARS is designed to assess the manifestations of ADHD in adults, and is composed of 42 items that are divided into four subscales including inattention/memory, hyperactivity/restlessness, impulsivity/emotional lability, and problems with self-concept^51^. Behavioral Activation System and Behavioral Inhibition System Questionnaires were used to measure self-reported dysregulation of behavioural activation and inhibition.

### Electroencephalogram (EEG) Acquisition and Analysis

During the EEG task, each participant was tested in a sound-attenuated EEG room. EEG was acquired using a NeuroScan SynAmps amplifier (Compumedics USA, E1 Paso, TX, USA) with 64 Ag-AgCl electrodes mounted on a Quik Cap using an extended 10–20 placement scheme. The ground electrode was located on the forehead and the physically linked reference electrode was attached to both mastoids. The vertical electrooculogram (EOG) was positioned above and below the left eye and the horizontal EOG was recorded at the outer canthus of each eye. The impedance was kept below 5 kΩ. All data were processed with a 0.1–100 Hz band pass filter and sampled at 1000 Hz.

The recorded EEG data were preprocessed using CURRY 7. Gross artifacts, such as artifacts caused by movements, were rejected through visual inspection by a trained person with no prior information regarding the origin of the data. Artifacts related to eye movement or eye blinks were removed using the mathematical procedure implemented in the preprocessing software^54^. The data were filtered using a 0.1–30 Hz bandpass filter and epoched from 100 ms pre-stimulus to 900 ms post-stimulus. The epochs were subtracted from the average value of the pre-stimulus interval for baseline correction. If any remaining epochs contained significant physiological artifacts (amplitude exceeding ±75 μV) in any of the 62 electrode sites, they were excluded from further analysis. Only artifact-free epochs were averaged across trials and participants for ERP analysis. For analysis of Go/Nogo task, only correctly responded epochs were used. The number of epochs of LDAEP used for the analysis did not significantly differ between the low and high LDAEP groups (60 dB: 185.22 ± 15.20 *vs*. 183.15 ± 16.29, *p* = 0.413, 70 dB: 184.41 ± 15.91 *vs.* 182.29 ± 17.30, *p* = 0.426, 80 dB: 184.27 ± 16.49 *vs.* 183.18 ± 16.27, *p* = 0.677, 90 dB: 185.38 ± 15.19 *vs.* 182.19 ± 17.13, *p* = 0.218, 100 dB = 184.47 ± 15.14 *vs.* 182.54 ± 17.17, *p* = 0.456). The number of epochs of Go/Nogo used for the analysis did not significantly differ between the low and high LDAEP groups (Go condition: 209.05 ± 21.37 *vs.* 208.35 ± 23.09, *p* = 0.845, Nogo condition: 49.37 ± 5.97 *vs.* 47.38 ± 7.34, *p* = 0.064).

#### Loudness dependence auditory evoked potentials (LDAEP)

Auditory stimulation included 1000 stimuli with an inter stimulus interval that was randomized between 500 and 900 ms. Tones of 1000 Hz and 80-ms duration (10-ms rise and 10-ms fall) were presented through MDR-D777 headphones (Sony, Tokyo, Japan) at five intensities: 60, 70, 80, 90, and 100 dB SPL. These stimuli were generated by E-Prime software (Psychology Software Tools, Pittsburgh, PA, USA). For each subject, the N1 peak (most negative peak between 50 and 200 ms from the stimulus) and the P2 peak (most positive peak between 150 and 300 ms from the stimulus) were then determined at the Cz electrode^55^,^56^ for the five intensities. The peak-to-peak N1/P2 amplitudes were calculated for the five stimulus intensities and the LDAEP was calculated as the slope of the linear regression.

#### Go/Nogo experiment

Subjects were seated approximately 60 cm away from a computer screen (Mitsubishi, 22-inch CRT monitor). Stimuli for Go/Nogo task, which consisted of numbers 1–8, were randomly presented on the screen. The subjects were instructed to press the spacebar as accurately and quickly as possible when the Go stimuli (even numbers: 2, 4, 6, 8) appeared at the centre of the screen and not to respond when the Nogo stimuli (odd numbers: 1, 3, 5, 7) were displayed. There were 300 trials, which consisted of Go (80% probability) condition and Nogo (20% probability) condition. On each task trial, a fixation cross was presented for 100 ms. Following intervals of 700–1000 ms, Go or Nogo targets appeared for 500 ms, and then, there was a 500 ms interval before the next trial. These stimuli were generated by E-Prime software (Psychology Software Tools, Pittsburgh, PA, USA). In the Go condition, the N200 (the most negative peak between 150 and 350 ms after stimulus onset) and the P300 (the most positive peak between 250 and 500 ms after stimulus onset) were investigated at the Fz, FCz, Cz, and Pz electrodes. In the Nogo condition, the N200 (the most negative peak between 150 and 350 ms after stimulus onset) and the P300 (the most positive peak between 300 and 550 ms after stimulus onset) were investigated at the Fz, FCz, Cz, and Pz electrodes.

### Source activity analysis

Standardized low-resolution brain electromagnetic tomography (sLORETA) is one of the representative source imaging methods for solving EEG inverse problem^57^. sLORETA assumes that the source activation of a voxel is similar to that of the surrounding voxels for calculating a particular solution, and applies an appropriate standardization of the current density. sLORETA was used to compute the cortical distribution of the standardized source current density of each ERP component. The lead field matrix was computed using a realistic head model segmented based on the Montreal Neurological Institute (MNI) 152 standard template, in which the three-dimensional solution space was restricted only to the cortical gray matter and hippocampus^58^. The solution space was composed of 6,239 voxels with 5 mm resolution. Anatomical labels such as Brodmann areas (BAs) are provided by using an appropriate transformation from MNI to Talairach space^59^.

The source images of N2 and P3 were analyzed in Nogo condition, and the time frames used to calculate the N2 and P3 source images were defined between 150 and 350 ms and between 300 and 550 ms after stimulus onset, respectively.

### Statistical Analysis

Multivariate ANOVA (MANOVA) was used to compare the scores of psychological and behavioural data between low and high LDAEP groups. A repeated measures analysis of variance (ANOVA) was performed for Go/Nogo ERP amplitudes and latencies, with the condition (Go and Nogo) and electrode site (Fz, FCz, Cz, and Pz) as the within-group factors, and the two comparing groups (low LDAEP vs. high LDAEP) as the between-group factor. Because LDAEP could be significantly influenced by gender, age, and smoking^60^, gender and age were considered as covariates in both the MANOVA and repeated measures ANOVA.

The comparison of sLORETA images between the two groups for Nogo-N2 and P3 was done using a statistical non-parametric mapping method (SnPM) that was provided by the sLORETA software. This software provides voxel-by-voxel independent t-test for the 6239 voxels, followed by a randomization test (n = 5000) to correct for multiple comparisons.

In addition, the relationships among variables were analyzed by Spearman’s correlation. When LDAEP was included in the correlation analysis, partial Spearman’s correlation was used to control age and sex as covariates. The significant level was set at *p* < 0.05 (two-tailed). Statistical analyses were performed using SPSS 21 (SPSS, Inc., Chicago, IL, USA).

## Additional Information

**How to cite this article**: Kim, J. S. *et al*. Auditory evoked potential could reflect emotional sensitivity and impulsivity. *Sci. Rep.* **6**, 37683; doi: 10.1038/srep37683 (2016).

**Publisher's note:** Springer Nature remains neutral with regard to jurisdictional claims in published maps and institutional affiliations.

## Figures and Tables

**Figure 1:**
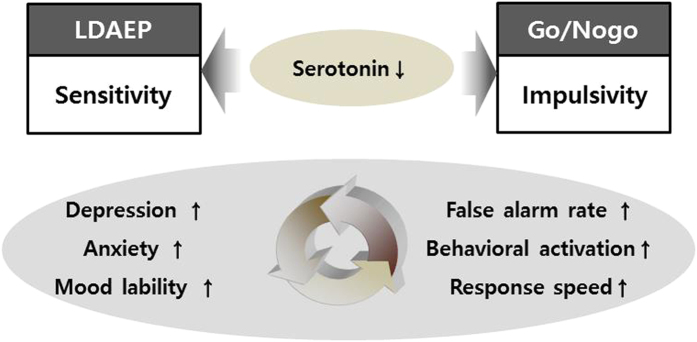
Study hypothesis. Loudness dependence of the auditory evoked potential (LDAEP) could reflect sensitivity and Go/Nogo reflects impulsivity, and both have shared serotonin related regulation. We hypothesize that LDAEP might be correlated with emotional sensitivity such as depression, anxiety, and mood lability. Moreover, LDAEP would be correlated with impulsivity, which would reflect response error, and fast speed.

**Table 1:** Comparison of baseline demographic, psychological, and behavioural characteristics in participants with low and high loudness dependence of the auditory evoked potential (LDAEP) (N = 157, low = 78, high = 79).

	Low LDAEP	High LDAEP	*p*
	*Mean* ± *SD* or N (%)		
Age (years)	27.33 ± 6.16	28.25 ± 6.59	0.45
Sex			
Male	36 (46.2)	21 (26.6)	**0.01**
Female	42 (53.8)	58 (73.4)	
Education (years)	14.40 ± 1.77	14.44 ± 1.77	0.77
Go reaction time (ms)	376.55 ± 25.45	375.65 ± 25.57	0.34
Go hit rate	0.94 ± 0.07	0.93 ± 0.08	0.78
Nogo false alarm rate	0.11 ± 0.08	0.15 ± 0.11	**0.01**
Barratt Impulsivity Scale (BIS)	58.10 ± 9.34	61.11 ± 9.10	**0.04**
Attentional impulsivity	15.58 ± 3.14	16.72 ± 3.71	**0.04**
Motor impulsivity	24.37 ± 4.95	25.96 ± 4.20	**0.05**
Non-planning impulsivity	18.15 ± 3.54	18.43 ± 3.99	0.50
State Anxiety Inventory (SAI)	35.58 ± 8.61	37.61 ± 7.35	0.16
Trait Anxiety Inventory (TAI)	38.12 ± 10.05	41.16 ± 9.55	0.09
Beck Depression Inventory (BDI)	6.81 ± 5.48	8.84 ± 5.90	0.06
Behavioral Activation System	36.50 ± 7.29	34.59 ± 3.90	0.10
Behavioral Inhibition System	21.28 ± 2.53	21.29 ± 1.99	0.69
Conners’ Adult ADHD rating scale (CAAR)	73.42 ± 13.94	76.90 ± 15.42	0.26
Inattention/Memory	23.95 ± 6.31	25.57 ± 6.85	0.21
Hyperactivity restlessness	17.91 ± 4.65	18.73 ± 4.73	0.25
Impulsivity/Emotional lability	17.46 ± 4.11	18.92 ± 4.66	**0.04**
Problem with self-concept	14.10 ± 3.08	13.67 ± 2.87	0.35
Affective Lability Scale (ALS)	13.56 ± 10.14	18.58 ± 9.25	**0.01**
Depression/Elation	5.46 ± 4.12	7.75 ± 3.97	**0.003**
Anxiety/Depression	4.86 ± 4.46	6.48 ± 4.29	0.07
Anger	3.24 ± 2.60	4.35 ± 2.35	**0.01**

**Table 2:** Brain regions showing significant differences of Nogo-P3 source activity between low and high LDAEP groups.

ROI (structure)	BA	MNI coordinates			Talairach coordinates			*t*
		X	Y	Z	X	Y	Z	
Cuneus	17	−10	−85	5	−10	−82	9	4.15*
Lingual Gyrus	17	−10	−90	0	−10	−87	4	4.48**
Precentral Gyrus	6	−40	−10	35	−40	−8	33	3.91*

Voxels showing maximum difference in the same structure are listed. Source activity of the listed areas was significantly increased in high LDAEP group.

^**^p < 0.01; ^*^p < 0.05.

## References

[b1] Seo D., Patrick C. J. & Kennealy P. J. Role of Serotonin and Dopamine System Interactions in the Neurobiology of Impulsive Aggression and its Comorbidity with other Clinical Disorders. Aggress Violent Behav 13, 383–395, doi: 10.1016/j.avb.2008.06.003 (2008). 19802333 PMC2612120

[b2] van Zutphen L., Siep N., Jacob G. A., Goebel R. & Arntz A. Emotional sensitivity, emotion regulation and impulsivity in borderline personality disorder: a critical review of fMRI studies. Neurosci Biobehav Rev 51, 64–76, doi: 10.1016/j.neubiorev.2015.01.001 (2015). 25616185

[b3] Hegerl U., Gallinat J. & Juckel G. Event-related potentials. Do they reflect central serotonergic neurotransmission and do they predict clinical response to serotonin agonists? J Affect Disord 62, 93–100 (2001). 11172876 10.1016/s0165-0327(00)00353-0

[b4] Juckel G., Molnar M., Hegerl U., Csepe V. & Karmos G. Auditory-evoked potentials as indicator of brain serotonergic activity–first evidence in behaving cats. Biol Psychiatry 41, 1181–1195 (1997). 9171909 10.1016/s0006-3223(96)00240-5

[b5] Hegerl U. & Juckel G. Intensity dependence of auditory evoked potentials as an indicator of central serotonergic neurotransmission: a new hypothesis. Biol Psychiatry 33, 173–187 (1993). 8383545 10.1016/0006-3223(93)90137-3

[b6] Fitzgerald P. B. . A study of intensity dependence of the auditory evoked potential (IDAEP) in medicated melancholic and non-melancholic depression. J Affect Disord 117, 212–216, doi: 10.1016/j.jad.2009.01.009 (2009). 19201033

[b7] Lee S. H., Sung K., Lee K. S., Moon E. & Kim C. G. Mismatch negativity is a stronger indicator of functional outcomes than neurocognition or theory of mind in patients with schizophrenia. Prog Neuropsychopharmacol Biol Psychiatry 48, 213–219, doi: 10.1016/j.pnpbp.2013.10.010 (2014). 24161665

[b8] Jagiellowicz J. . The trait of sensory processing sensitivity and neural responses to changes in visual scenes. Social cognitive and affective neuroscience 6, 38–47, doi: 10.1093/scan/nsq001 (2011). 20203139 PMC3023077

[b9] Brown S. M., Manuck S. B., Flory J. D. & Hariri A. R. Neural basis of individual differences in impulsivity: contributions of corticolimbic circuits for behavioral arousal and control. Emotion (Washington, D.C.) 6, 239–245, doi: 10.1037/1528-3542.6.2.239 (2006). 16768556

[b10] Smith J. L., Mattick R. P., Jamadar S. D. & Iredale J. M. Deficits in behavioural inhibition in substance abuse and addiction: a meta-analysis. Drug Alcohol Depend 145, 1–33, doi: 10.1016/j.drugalcdep.2014.08.009 (2014). 25195081

[b11] Bruin K. J., Wijers A. A. & van Staveren A. S. Response priming in a go/nogo task: do we have to explain the go/nogo N2 effect in terms of response activation instead of inhibition? Clin Neurophysiol 112, 1660–1671 (2001). 11514249 10.1016/s1388-2457(01)00601-0

[b12] Falkenstein M., Hoormann J. & Hohnsbein J. ERP components in Go/Nogo tasks and their relation to inhibition. Acta Psychol (Amst) 101, 267–291 (1999). 10344188 10.1016/s0001-6918(99)00008-6

[b13] Nieuwenhuis S., Yeung N., van den Wildenberg W. & Ridderinkhof K. R. Electrophysiological correlates of anterior cingulate function in a go/no-go task: effects of response conflict and trial type frequency. Cogn Affect Behav Neurosci 3, 17–26 (2003). 12822595 10.3758/cabn.3.1.17

[b14] Dimoska A. & Johnstone S. J. Neural mechanisms underlying trait impulsivity in non-clinical adults: stop-signal performance and event-related potentials. Prog Neuropsychopharmacol Biol Psychiatry 31, 443–454, doi: 10.1016/j.pnpbp.2006.11.009 (2007). 17175083

[b15] Bohne A., Savage C. R., Deckersbach T., Keuthen N. J. & Wilhelm S. Motor inhibition in trichotillomania and obsessive-compulsive disorder. J Psychiatr Res 42, 141–150, doi: 10.1016/j.jpsychires.2006.11.008 (2008). 17215004

[b16] Buchmann J., Gierow W., Reis O. & Haessler F. Intelligence moderates impulsivity and attention in ADHD children: an ERP study using a go/nogo paradigm. World J Biol Psychiatry 12 Suppl 1, 35–39, doi: 10.3109/15622975.2011.600354 (2011). 21905993

[b17] Guan M. . Impaired response inhibition in juvenile delinquents with antisocial personality characteristics: A preliminary ERP study in a Go/Nogo task. Neurosci Lett 603, 1–5, doi: 10.1016/j.neulet.2015.06.062 (2015). 26189594

[b18] Messerotti Benvenuti S., Sarlo M., Buodo G., Mento G. & Palomba D. Influence of impulsiveness on emotional modulation of response inhibition: An ERP study. Clin Neurophysiol 126, 1915–1925, doi: 10.1016/j.clinph.2014.12.012 (2015). 25595704

[b19] Beste C., Domschke K., Radenz B., Falkenstein M. & Konrad C. The functional 5-HT1A receptor polymorphism affects response inhibition processes in a context-dependent manner. Neuropsychologia 49, 2664–2672, doi: 10.1016/j.neuropsychologia.2011.05.014 (2011). 21645534

[b20] Macoveanu J. . Serotonin 2A receptors, citalopram and tryptophan-depletion: a multimodal imaging study of their interactions during response inhibition. Neuropsychopharmacology 38, 996–1005, doi: 10.1038/npp.2012.264 (2013). 23303045 PMC3629389

[b21] Eagle D. M., Bari A. & Robbins T. W. The neuropsychopharmacology of action inhibition: cross-species translation of the stop-signal and go/no-go tasks. Psychopharmacology (Berl) 199, 439–456, doi: 10.1007/s00213-008-1127-6 (2008). 18542931

[b22] Uhl I. . Loudness dependence of auditory evoked potentials (LDAEP) in clinical monitoring of suicidal patients with major depression: a pilot study. Eur Arch Psychiatry Clin Neurosci 262, 487–492, doi: 10.1007/s00406-012-0297-8 (2012). 22350533

[b23] Omura K. & Kusumoto K. Sex differences in neurophysiological responses are modulated by attentional aspects of impulse control. Brain Cogn 100, 49–59, doi: 10.1016/j.bandc.2015.09.006 (2015). 26473554

[b24] Zhang B. W., Zhao L. & Xu J. Electrophysiological activity underlying inhibitory control processes in late-life depression: a Go/Nogo study. Neurosci Lett 419, 225–230, doi: 10.1016/j.neulet.2007.04.013 (2007). 17462822

[b25] Lee S. H., Park Y. C., Yoon S., Kim J. I. & Hahn S. W. Clinical implications of loudness dependence of auditory evoked potentials in patients with atypical depression. Prog Neuropsychopharmacol Biol Psychiatry 54, 7–12, doi: 10.1016/j.pnpbp.2014.05.010 (2014). 24865151

[b26] Hartmann L., Sallard E. & Spierer L. Enhancing frontal top-down inhibitory control with Go/NoGo training. Brain Struct Funct, doi: 10.1007/s00429-015-1131-7 (2015). 26459141

[b27] Liu Q., Zhou R., Liu L. & Zhao X. Effects of 72hours total sleep deprivation on male astronauts’ executive functions and emotion. Compr Psychiatry 61, 28–35, doi: 10.1016/j.comppsych.2015.05.015 (2015). 26112064

[b28] Reynolds B., Penfold R. B. & Patak M. Dimensions of impulsive behavior in adolescents: laboratory behavioral assessments. Exp Clin Psychopharmacol 16, 124–131, doi: 10.1037/1064-1297.16.2.124 (2008). 18489016

[b29] Dong G., Zhou H. & Zhao X. Impulse inhibition in people with Internet addiction disorder: electrophysiological evidence from a Go/NoGo study. Neurosci Lett 485, 138–142, doi: 10.1016/j.neulet.2010.09.002 (2010). 20833229

[b30] Ruchsow M. . Impulsiveness and ERP components in a Go/Nogo task. J Neural Transm (Vienna) 115, 909–915, doi: 10.1007/s00702-008-0042-7 (2008). 18368285

[b31] Lansbergen M. M., Bocker K. B., Bekker E. M. & Kenemans J. L. Neural correlates of stopping and self-reported impulsivity. Clin Neurophysiol 118, 2089–2103, doi: 10.1016/j.clinph.2007.06.011 (2007). 17652017

[b32] Boterberg S. & Warreyn P. Making sense of it all: The impact of sensory processing sensitivity on daily functioning of children. Personality and Individual Differences 92, 80–86 (2016).

[b33] Smolewska K. A., McCabe S. B. & Woody E. Z. A psychometric evaluation of the Highly Sensitive Person Scale: The components of sensory-processing sensitivity and their relation to the BIS/BAS and “Big Five”. Personality and Individual Differences 40, 1269–1279 (2006).

[b34] Rohl M. & Uppenkamp S. An auditory fMRI correlate of impulsivity. Psychiatry Res 181, 145–150, doi: 10.1016/j.pscychresns.2009.09.002 (2010). 20083394

[b35] Juckel G., Schmidt L. G., Rommelspacher H. & Hegerl U. The Tridimensional Personality Questionnaire and the intensity dependence of auditory evoked dipole source activity. Biol Psychiatry 37, 311–317, doi: 10.1016/0006-3223(94)00118-M (1995). 7748982

[b36] Konishi S., Nakajima K., Uchida I., Sekihara K. & Miyashita Y. No-go dominant brain activity in human inferior prefrontal cortex revealed by functional magnetic resonance imaging. Eur J Neurosci 10, 1209–1213 (1998). 9753190 10.1046/j.1460-9568.1998.00167.x

[b37] Menon V., Adleman N. E., White C. D., Glover G. H. & Reiss A. L. Error-related brain activation during a Go/NoGo response inhibition task. Hum Brain Mapp 12, 131–143 (2001). 11170305 10.1002/1097-0193(200103)12:3<131::AID-HBM1010>3.0.CO;2-C PMC6872006

[b38] Sasaki K. & Gemba H. Electrical activity in the prefrontal cortex specific to no-go reaction of conditioned hand movement with colour discrimination in the monkey. Exp Brain Res 64, 603–606 (1986). 3803495 10.1007/BF00340499

[b39] Smith J. L., Jamadar S., Provost A. L. & Michie P. T. Motor and non-motor inhibition in the Go/NoGo task: an ERP and fMRI study. Int J Psychophysiol 87, 244–253, doi: 10.1016/j.ijpsycho.2012.07.185 (2013). 22885679

[b40] Braet W. . Functional developmental changes underlying response inhibition and error-detection processes. Neuropsychologia 47, 3143–3151, doi: 10.1016/j.neuropsychologia.2009.07.018 (2009). 19651151

[b41] Gothelf D. . Abnormal cortical activation during response inhibition in 22q11.2 deletion syndrome. Hum Brain Mapp 28, 533–542, doi: 10.1002/hbm.20405 (2007). 17427209 PMC6871340

[b42] Nakata H. . Somato-motor inhibitory processing in humans: an event-related functional MRI study. Neuroimage 39, 1858–1866, doi: 10.1016/j.neuroimage.2007.10.041 (2008). 18083602

[b43] Lovie-Kitchin J. E. Validity and reliability of visual acuity measurements. Ophthalmic Physiol Opt 8, 363–370 (1988). 3253626 10.1111/j.1475-1313.1988.tb01170.x

[b44] Rhee M. K. . A standardization study of Beck depression inventory I; Korean version (K-BDI): Reliability and factor analysis. The Korean Journal of Psychopathology 4, 77–95 (1995).

[b45] Kim J. T. & Shin D. K. A study based on the standardization of the STAI for Korea. Recent Medicine 2, 69–75 (1978).

[b46] Harvey P. D., Greenberg B. R. & Serper M. R. The affective lability scales: development, reliability, and validity. Journal of clinical psychology 45, 786–793 (1989). 2808736 10.1002/1097-4679(198909)45:5<786::aid-jclp2270450515>3.0.co;2-p

[b47] Oh J. H. *Validation of the Affective Lability Scales in a Korean population* Masters thesis, Dongduk, (2014).

[b48] Aas M. . Psychometric properties of the Affective Lability Scale (54 and 18-item version) in patients with bipolar disorder, first-degree relatives, and healthy controls. J Affect Disord 172, 375–380, doi: 10.1016/j.jad.2014.10.028 (2015). 25451440

[b49] Lee S. R. . The Study on Reliability and Validity of Korean Version of the Barratt Impulsiveness Scale-11-Revised in Nonclinical Adult Subjects. J Korean Neuropsychiatr Assoc 51, 378–386 (2012).

[b50] Patton J. H., Stanford M. S. & Barratt E. S. Factor structure of the Barratt impulsiveness scale. Journal of clinical psychology 51, 768–774 (1995). 8778124 10.1002/1097-4679(199511)51:6<768::aid-jclp2270510607>3.0.co;2-1

[b51] Park J. S., Lee W. H., Lee S. R., Kim S. M. & Bahn G. H. Reliability and Validity of Korean Version of the Conners Adult Attention Deficit Hyperactivity Disorder Scale in General Population. J Korean Neuropsychiatr Assoc (2013).

[b52] Carver C. S. & White T. L. Behavioral inhibition, behavioral activation, and affective responses to impending reward and punishment: The BIS/BAS scales. Journal of Personality and Social Psychology 67, 319–333 (1994).

[b53] Kim K. H. & Kim W. S. Korean-BAS/BIS Scale. Korean J Health Psychol 6, 19–37 (2001).

[b54] Semlitsch H. V., Anderer P., Schuster P. & Presslich O. A solution for reliable and valid reduction of ocular artifacts, applied to the P300 ERP. Psychophysiology 23, 695–703 (1986). 3823345 10.1111/j.1469-8986.1986.tb00696.x

[b55] Gudlowski Y. . Serotonergic dysfunction in the prodromal, first-episode and chronic course of schizophrenia as assessed by the loudness dependence of auditory evoked activity. Schizophr Res 109, 141–147, doi: 10.1016/j.schres.2009.02.008 (2009). 19268544

[b56] Park Y. M., Jung E., Kim H. S., Hahn S. W. & Lee S. H. Differences in central serotoninergic transmission among patients with recent onset, sub-chronic, and chronic schizophrenia as assessed by the loudness dependence of auditory evoked potentials. Schizophr Res 168, 180–184, doi: 10.1016/j.schres.2015.07.036 (2015). 26232871

[b57] Pascual-Marqui R. D. Standardized low-resolution brain electromagnetic tomography (sLORETA): technical details. Methods Find Exp Clin Pharmacol 24 Suppl D, 5–12 (2002). 12575463

[b58] Fuchs M., Kastner J., Wagner M., Hawes S. & Ebersole J. S. A standardized boundary element method volume conductor model. Clin Neurophysiol 113, 702–712 (2002). 11976050 10.1016/s1388-2457(02)00030-5

[b59] Brett M., Johnsrude I. S. & Owen A. M. The problem of functional localization in the human brain. Nat Rev Neurosci 3, 243–249, doi: 10.1038/nrn756 (2002). 11994756

[b60] Min J. A. . Clinical characteristics associated with different strengths of loudness dependence of auditory evoked potentials (LDAEP) in major depressive disorder. Psychiatry Res 200, 374–381, doi: 10.1016/j.psychres.2012.06.038 (2012). 23021319

